# Nrf2 inhibits ferroptosis and protects against acute lung injury due to intestinal ischemia reperfusion via regulating SLC7A11 and HO-1

**DOI:** 10.18632/aging.103378

**Published:** 2020-06-29

**Authors:** Hui Dong, Zhuanzhuan Qiang, Dongdong Chai, Jiali Peng, Yangyang Xia, Rong Hu, Hong Jiang

**Affiliations:** 1 Shanghai Ninth People’s Hospital, Shanghai JiaoTong University School of Medicine, Centre for Specialty Strategy Research of Shanghai Jiao Tong University China Hospital Development Institute, Shanghai 200011, China

**Keywords:** ferroptosis, acute lung injury, solute carrier family 7 member 11, heme oxygenase-1, uclear factor erythroid 2 related factor2

## Abstract

Acute lung injury (ALI) is a syndrome associated with a high mortality rate. Nrf2 is a key regulator of intracellular oxidation homeostasis that plays a pivotal role in controlling lipid peroxidation, which is closely related to the process of ferroptosis. However, the intrinsic effect of Nrf2 on ferroptosis remains to be investigated in ALI. We found that MDA expression increased while GSH and GPX4 decreased in ALI models. Furthermore, the characteristic mitochondrial morphological changes of ferroptosis appear in type II alveolar epithelial cells in IIR models. Additional pre-treatment of Fe and Ferrostatin-1 in ALI significantly aggravated or ameliorated the pathological injuries of lung tissue, pulmonary edema, lipid peroxidation, as well as promoted or prevented cell death, respectively. Knocking down Nrf2 notably decreased the expression of SLC7A11 and HO-1. Interference with SLC7A11 markedly increased Nrf2-HO-1 and dramatically attenuated cell death in OGD/R models. These findings indicate that ferroptosis can be inhibited by Nrf2 through regulating SLC7A11 and HO-1, which may provide a potential therapeutic strategy for IIR-ALI.

## INTRODUCTION

Intestinal ischemia/reperfusion (IIR) injury is an intraoperative complication that can give rise to secondary vascular disease and a number of associated physiological conditions, which are predominantly related to the release of cytotoxic substances from ischemic tissues and inflammatory mediators [1]. As a consequence, IIR may lead to sepsis, systemic inflammatory response syndrome, and multiple organ dysfunction syndrome [2]. Moreover, IIR has also been shown to cause acute lung injury (ALI), which in turn leads to a condition termed acute respiratory distress syndrome and contributes to the high mortality associated with IIR [3–5]. Thus, ALI caused by IIR is a challenging concern in the field of critical illness research [6].

Ferroptosis was initially reported in 2012 by Dixon et al [7, 8]. Ferroptosis can directly or indirectly inhibit glutathione peroxidase 4 (GPX4), which leads to intracellular antioxidant system damage and reactive oxygen species (ROS) accumulation in mitochondria, thereby causing cellular dysfunction [9, 10]. It was found that increasing cellular ferroptosis could further aggravate the functional damage to the viscera in models of renal and liver injury induced by ischemia-reperfusion [11, 12]. However, the molecular mechanism of ferroptosis in ALI following ischemia reperfusion has not yet been reported.

Nuclear factor E2 related factor 2 (Nrf2) is the key regulatory factor required for cells to maintain an oxidative steady state and is activated under conditions of high oxidative stress. Nrf2 can then promote target gene transcription, as well as antioxidant and anti-inflammatory protein translation by combining to the antioxidant response element (ARE) in the nucleus, which promotes cellular protection [13]. Studies have previously reported that HO-1 plays a role in antioxidant protection under a state of high oxidative stress via control of the Nrf2/ARE pathway [14].

The SLC7A11 gene belongs to the solute transport family and encodes a cystine/glutamate xCT transporter, a key gene involved in regulating “iron overload-ferroptosis” [15]. In addition, Lin et al. [16] found that down-regulating *SLC7A11* expression can result in decreased cystine-dependent glutathione peroxidase activity, cellular antioxidant capacity, lipid activity and oxygen elevation, which eventually causes cellular ferroptosis.

In the present study, we investigated the features of ferroptosis in ALI following IIR and whether Nrf2 regulates ferroptosis and protects against ALI. To this end, we used C57BL/6 and Nrf2 gene knockout mice to establish an IIR-ALI model. The results clearly show that ferroptosis occurs in this model of ALI following IIR, and Nrf2 can regulate ferroptosis and protect against ALI.

## RESULTS

### Ferroptosis occurs in ALI due to IIR

**Figure 1:**
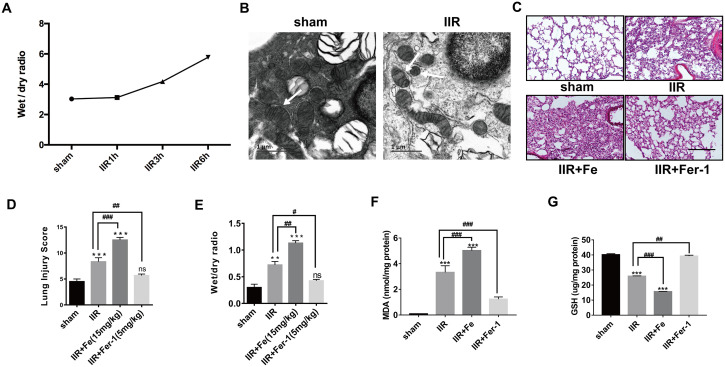
**IIR induces ferroptosis in type II alveolar epithelial cells.** (**A**) The degree of pulmonary oedema continued to increase with the extension of reperfusion time. (**B**) Representative transmission electron micrographs of the ultrastructure of lung tissues. Scale bars: 1 μm. (**C**) HE staining of the lung tissues of mice following IIR, IIR + Fe, and IIR + Fer-1. Scale bars: 200 μm. (**D**) The lung pathological damage score showed an addition and reduction after Fe and Fer-1 administration, respectively. (**E**) The wet to dry ratio of the lung tissue shown in each group. (**F**) The level of lipid peroxide MDA in each group. (**G**) The GSH level in each group. The error bars represent the standard error from three replicates. Data are presented as the mean ± SEM. *P < 0.05; **P < 0.01; ***P < 0.001 between the groups; *compared with the sham group; # compared with the IIR group.

To first verify the reliability of the model, we established that the level of pulmonary tissue oedema gradually worsened with prolonged IIR time (Figure 1A). We next explored the role of ferroptosis in the IIR-ALI model. Using transmission electron microscopy, we observed that compared with the sham group, the mitochondrial morphology in type II alveolar epithelial cells of mice in the IIR group showed the characteristic changes of ferroptosis, including the presence of smaller mitochondria and reduced cristae (Figure 1B). Through HE staining of pathological sections (Figure 1C) and W/D experiments (Figure 1E), we found that after IIR, the extent of lung tissue damage was aggravated, and the degree of pulmonary oedema had increased (*P* < 0.05). In addition, the lipid peroxide MDA (Figure 1F) increased while the reduced glutathione (GSH) (Figure 1G) decreased, which are characteristic indicators of ferroptosis. To this end, we injected Fe (a promoter of ferroptosis) and ferrostatin-1 (Fer-1, a specific ferroptosis inhibitor) into the tail veins of mice. The lung tissue was collected from the mice after completing the experiment. Pathological HE staining (Figure 1C) and the wet to dry ratios of the lung tissues (Figure 1E) showed a larger area of inflammatory cell infiltration in the alveoli or more severe pulmonary oedema following the addition of Fe. In addition, the extent of the injury was reduced compared with the IIR group following Fer-1 administration. Similarly, the level of MDA (Figure 1F) was further increased or decreased with the application of Fe or ferrostatin-1 respectively compared to the IIR group; however, the opposite was observed with the level of GSH (Figure 1G). These are all characteristic indicators of ferroptosis. Therefore, these findings suggest that ferroptosis occurred in IIR-ALI.

### The Nrf2-SLC7A11/HO-1 pathway can inhibit ferroptosis and play a protective role in IIR-ALI

**Figure 2:**
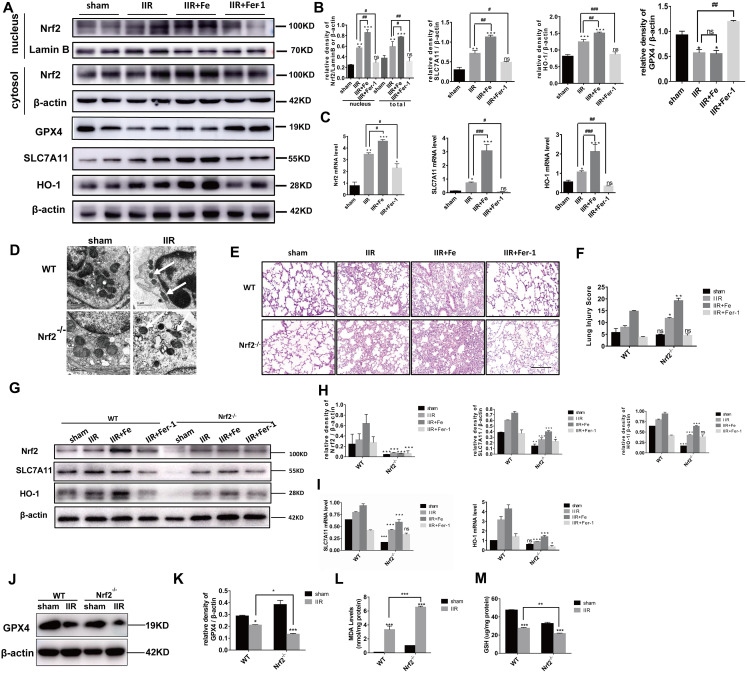
**Nrf2 regulates SLC7A11 and HO-1 to inhibit ferroptosis and protect against IIR-ALI.** (**A**) Western blot analysis of Nrf2, HO-1, SLC7A11, and GPX4 expression in the lung tissues of each group. (**B**) The representative quantification of these proteins. (**C**) Relative mRNA expression of Nrf2, SLC7A11, and HO-1 in each group. (**D**) Representative transmission electron micrographs of the ultrastructure of the lung tissues. Scale bars: 1 μm. (**E**) Representative HE-stained lung sections. Morphology was examined using light microscopy. Scale bars: 200 μm. (**F**) Pathological scores were assigned by an experienced pathologist. (**G**) Western blot analysis of Nrf2, SLC7A11, and HO-1 expression in the lung tissues of each group. (**H**) The representative quantification of these proteins. (**I**) Relative mRNA expression of SLC7A11 and HO-1 in each group. (**J**) Western blot analysis of GPX4 expression in each group. (**K**) The representative quantification of GPX4 protein. (**L**) The lipid peroxide MDA level in each group. (**M**) The GSH level in each group. The error bars represent the standard error from three replicates. Data are presented as the mean ± SEM. *P < 0.05; **P < 0.01;***P < 0.001. # compared with the IIR group in WT mice.

Following IIR-ALI, the transcriptional activity of Nrf2 increased, and cytoplasmic Nrf2 was transferred into the nucleus. The level of total Nrf2, SLC7A11, and HO-1 protein expression was enhanced, whereas the expression of the GPX4 was reduced. The first three proteins displayed enhanced severity following the administration of Fe. However, the level of protein expression was reduced compared with that of the OGD/R group following the administration of Fer-1. In contrast, the level of GPX4 protein expression could be increased by Fer-1 administration. (Figure 2A). The RT-PCR results were consistent with the Western blot (WB) analysis (Figure 2C). We used *Nrf2* gene knockout mice to further explore the role of Nrf2 in ferroptosis and IIR-ALI. The mitochondria observed via transmission electron microscopy had shrunk in size, the cristae had decreased or disappeared, and the outer membrane had ruptured in the Nrf2^-/-^ IIR group, suggesting that Nrf2 can alleviate ferroptosis (Figure 2D). Similarly, the extent of pathological damage in the Nrf2^-/-^ IIR group was more severe than that observed in WT mice (Figure 2E), suggesting that Nrf2 plays a protective role in ALI, which is also consistent with previous studies [17]. The damage observed in the IIR + Fe group was more severe than that of the corresponding group of WT mice, and the damage in the *Nrf2* knockout mice was also reduced following the administration of Fer-1 (Figure 2E). In addition, a WB was performed on the lung tissues of both *Nrf2* knockout and WT mice in each of the four groups. The results showed that the level of SLC7A11 and HO-1 expression was significantly reduced in the *Nrf2* knockout mice (Figure 2G). In addition, GPX4 expression in the IIR group of the *Nrf2* knockout mice was further decreased compared with the WT IIR group (Figure 2J). The RT-PCR results were consistent with the WB findings (Figure 2I). When the *Nrf2* gene was knocked out, MDA expression was further increased (Figure 2L), while GSH expression was decreased (Figure 2M) compared with the mice in the WT IIR group. These data indicate that Nrf2 regulates SLC7A11 and HO-1 to inhibit ferroptosis and plays a protective role in IIR-ALI.

### Oxygen-glucose deprivation and reoxygenation (OGD/R) induces ferroptotic cell death in MLE12 and BEAS-2B cells

**Figure 3:**
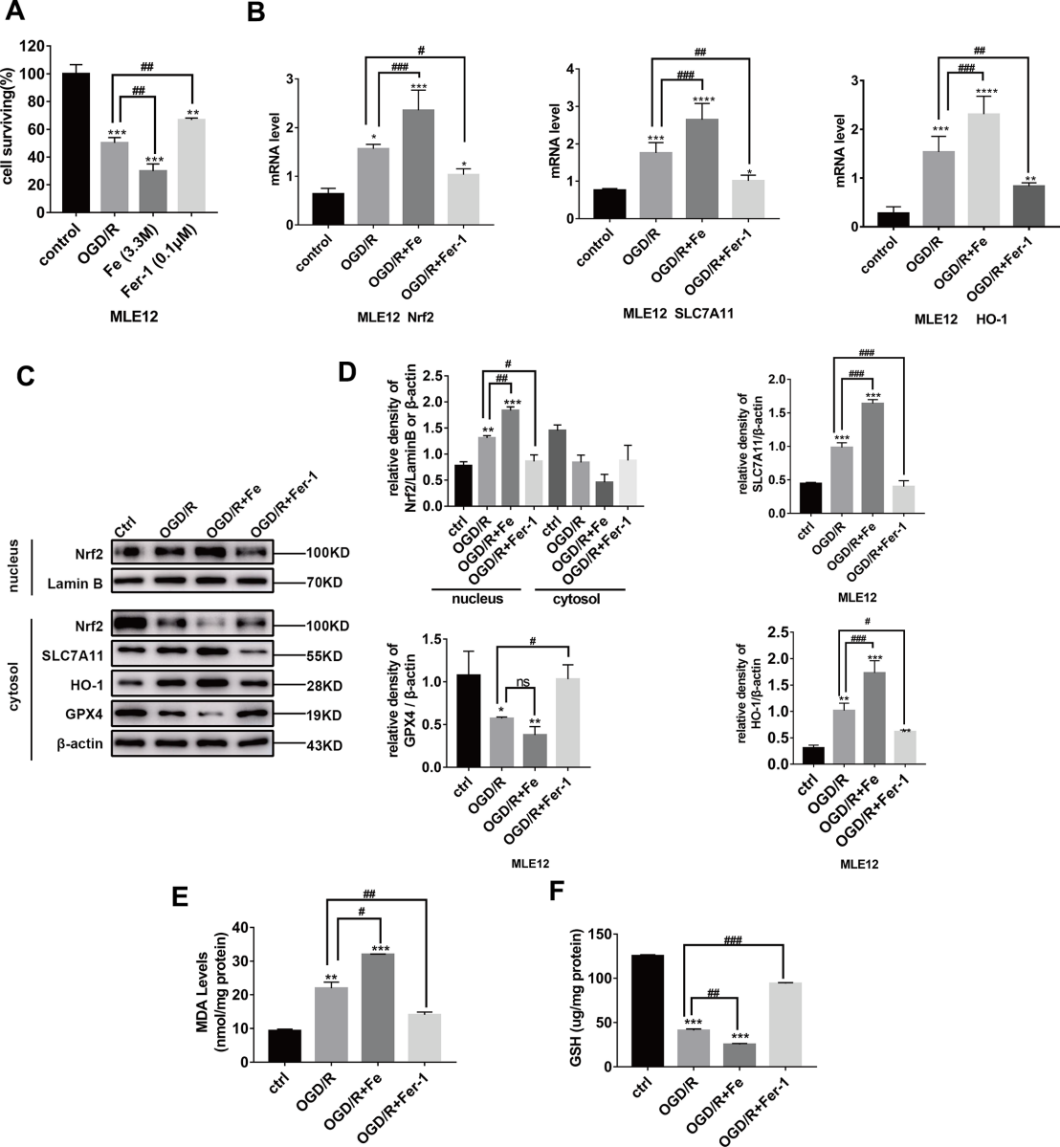
**OGD/R induces ferroptosis in pulmonary epithelial cells and increases the level of Nrf2 /SLC7A11/HO-1 expression during ferroptosis.** (**A**) The cells surviving after OGD (8 h)/R (12 h), while the administration of Fe (3.3M) (800 μg/mL)/Fer-1 (0.1 μM) can respectively increase or decrease the ratio. (**B**) Relative mRNA expression of Nrf2, HO-1, and SLC7A11 in MLE12. (**C**) Western blotting of Nrf2, HO-1, GPX4, and SLC7A11 protein expression in each group. (**D**) The representative quantification of these proteins. (**E**) The level of lipid peroxide MDA in each group. (**F**) The GSH level in each group. The error bars represent the standard error from three replicates. Data are presented as the mean ± SEM. *P < 0.05; **P < 0.01; ***P < 0.001; *compared with control; # compared with OGD/R.

To further confirm our initial findings, MLE12 and BEAS-2B cell lines were selected for in vitro experiments. Similar to previous findings, we verified the OGD/R model in vitro. OGD/R significantly decreased the number of adherent cells (all *P* < 0.001) (Figure 3A and Supplementary Figure 1A). Cell viability was further decreased with the addition of Fe (3.3 M), whereas this effect was markedly alleviated by the co-incubation of MLE12 and BEAS-2B cells with ferrostatin-1 (0.1 μM). Taken together, our findings suggest that OGD/R induces ferroptotic-mediated cell death in pulmonary epithelial cells.

### Nrf2/SLC7A11/HO-1 activation contributes to the resistance of pulmonary epithelial cells to OGD/R-induced ferroptosis

The level of Nrf2, SLC7A11, and HO-1 protein expression in MLE12 and BEAS-2B cells (Figure 3C and Supplementary Figure 1C) increased following OGD/R, and displayed enhanced severity following the administration of Fe (3.3 M). However, the level of protein expression was reduced compared with that of the OGD/R group following the administration of Fer-1 (0.1 μM). Similar findings were found for the changes in MDA levels (Figure 3E). In contrast, the cell survival rate (Figure 3A), GPX4 protein expression (Figure 3C), and GSH levels (Figure 3F) showed opposite changes, which are classic measurements of ferroptosis. The expression of Nrf2 in the nuclear extracts of cells after OGD/R with or without Fe or Fer–1 was markedly increased (Figure 3C and Supplementary Figure 1C). The RT-PCR results were consistent with the WB findings (Figure 3B and Supplementary Figure 1B). Together, these findings further confirm that OGD/R promotes the entry of Nrf2 into the nucleus and protects against OGD/R by inhibiting ferroptosis.

### Nrf2 alleviates OGD/R-induced ferroptosis through upregulating SLC7A11 and HO-1

**Figure 4:**
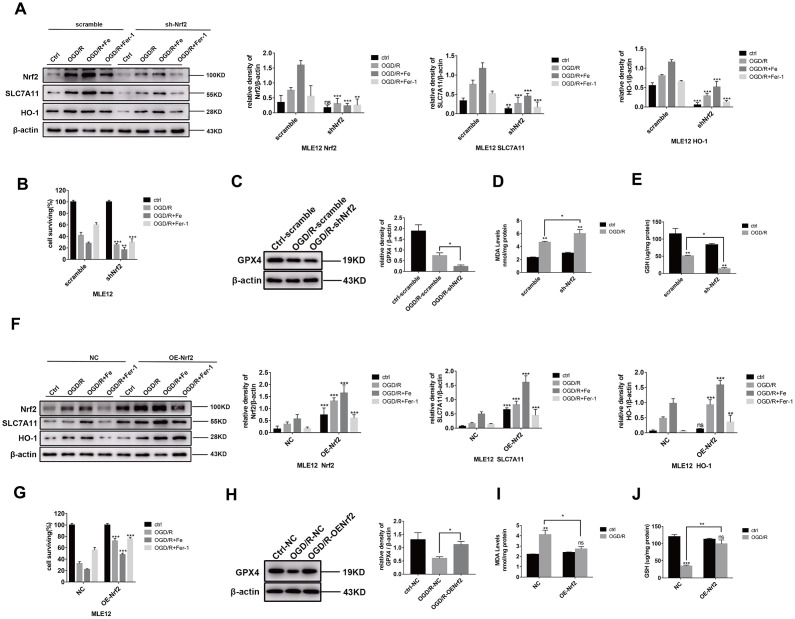
**Nrf2 activation contributes to ferroptosis resistance.** The cells were transfected with lentiviruses (**A** and **F**). Western blot analysis of the level of Nrf2, HO-1, and SLC7A11 protein expression in each group and the representative quantification of these proteins. (**B** and **G**) Cell viability was determined by a CCK-8 assay (n = 3). (**C** and **H**) Western blot analysis of the level of GPX4 protein expression in each group and the representative quantification of GPX4. (**D** and **I**) The level of lipid peroxide MDA in each group. (**E** and **J**) The level of GSH in each group. The error bars represent the standard error from three replicates. Data are presented as the mean ± SEM. *P < 0.05; **P < 0.01; ***P < 0.001.

To elucidate the underlying mechanisms by which Nrf2 exerts its function, MLE12 and BEAS-2B were transfected with an Nrf2-shRNA lentivirus. We found that the *Nrf2* knockdown downregulated the level of *SLC7A11* and *HO-1* gene expression (Figure 4A and Supplementary Figure 2A). The cell viability, GPX4 protein expression, and level of GSH decreased to a greater extent than the scrambled cells following exposure to OGD/R (Figure 4B, Supplementary Figure 2B, 4C, and 4E). In contrast, the level of MDA was higher than that of the scrambled cells following exposure to OGD/R (Figure 4D). Conversely, Nrf2 overexpression with a lentivirus significantly increased the level of *SLC7A11* and HO-1 protein expression (Figure 4F and Supplementary Figure 2C) and cell viability (Figure 4G and Supplementary Figure 2D). The level of GPX4 and GSH protein expression were also significantly increased (Figure 4H, and 4J). Moreover, the level of MDA (Figure 4I) decreased to a greater extent than that of the NC cells following exposure to OGD/R. Therefore, these findings suggest that Nrf2 alleviates OGD/R-induced ferroptosis by upregulating SLC7A11 and HO-1.

### SLC7A11 negatively regulates Nrf2-HO-1-mediated resistance to ferroptosis

**Figure 5:**
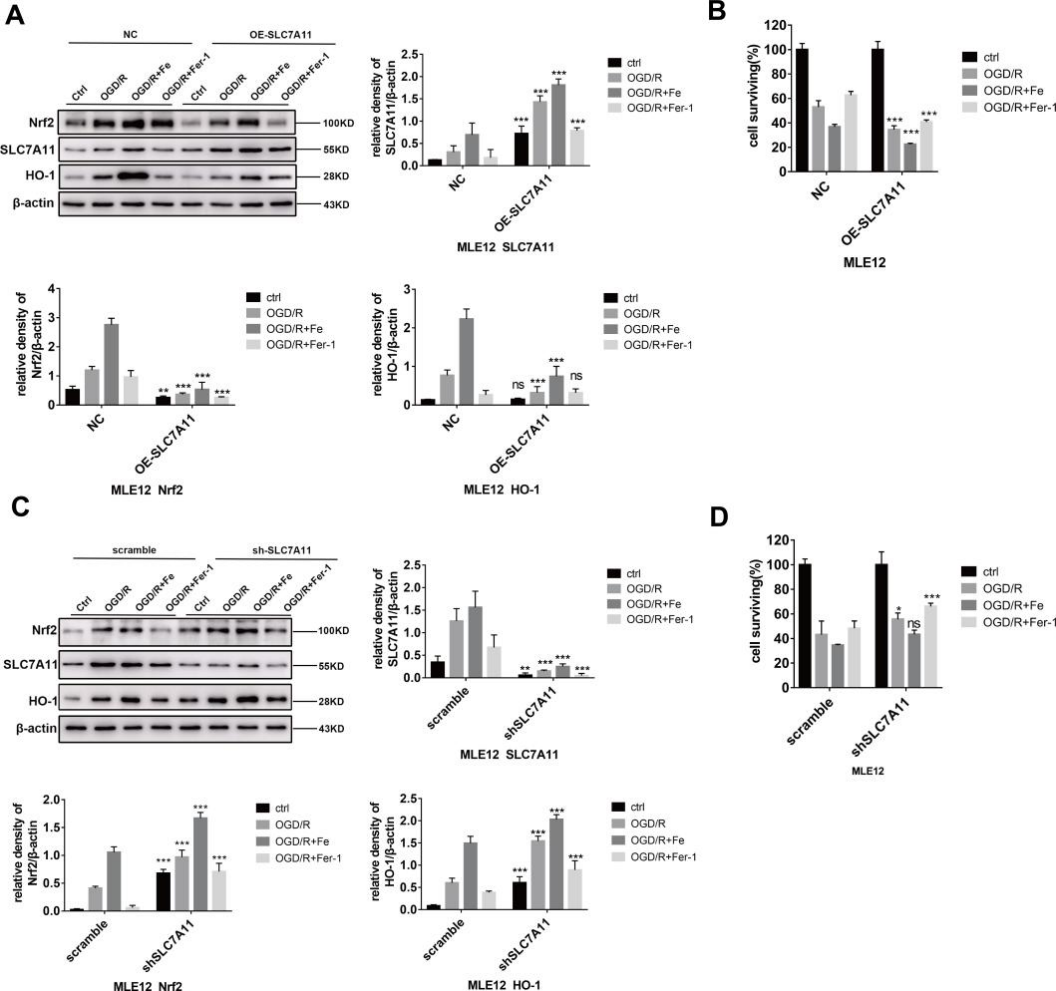
**Low levels of SLC7A11 alleviate cell death by upregulating Nrf2-HO-1, whereas SLC7A11 overexpression (OE-SLC7A11) enhanced cell death.** (**A** and **C**) Western blot analysis of the Nrf2, HO-1, and SLC7A11 in each group and the representative quantification of these proteins in MLE12 cells. (**B** and **D**) Cell viability was determined using a CCK-8 assay (n = 3). The error bars represent the standard error from three replicates. Data are presented as the mean ± SEM. *P < 0.05; **P < 0.01; ***P < 0.001.

Since both SLC7A11 and HO-1 are regulated by Nrf2 (i.e., they are located downstream of Nrf2), we further explored the interactions between SLC7A11 and HO-1. MLE12 and BEAS-2B were transfected with a *SLC7A11*-shRNA lentivirus or *SLC7A11*-overexpressing lentivirus. The results showed that SLC7A11 overexpression downregulated Nrf2-HO-1 (Figure 5A and Supplementary Figure 3A) and dramatically increased cell death as a result (Figure 5B and Supplementary Figure 3B). Conversely, SLC7A11 interference increased Nrf2-HO-1 expression (Figure 5C and Supplementary Figure 3C) and attenuated cell death (Figure 5D and Supplementary Figure 3D). Finally, HO-1 expression was inhibited by the HO-1 specific inhibitor, Snpp (Tin protoporphyrin IX), to observe the level of SLC7A11 protein expression. As expected, SLC7A11 expression did not change significantly following HO-1 inhibition (Supplementary Figure 3E).

## DISCUSSION

Ferroptosis was identified as a new form of regulated cell death [18] characterized by lipid peroxidation and is strongly dependent on both iron and ROS accumulation. Since increasing evidence suggests that reactive oxygen species (ROS) play a key role in the pathogenesis of intestinal ischemia–reperfusion, we hypothesized that IIR or OGD/R may be an important process in facilitating the occurrence of ferroptosis [19]. To validate this hypothesis, we examined the levels of GPX4 expression, as well as the GSH and MDA content in the lung tissues and MLE12 cells after IIR or OGD/R. In the above models, the expression of proferroptotic factors increased while that of antiferroptotic factors decreased. At the same time, the characteristic mitochondrial morphological changes associated with ferroptosis appeared in type II alveolar epithelial cells in the IIR mice, indicating that the epithelium might experience ferroptosis during IIR. To pursue the effect of ferroptosis on IIR-ALI, Fe and ferrostatin-1 were injected through the tail veins of mice. As a special inhibitor of ferroptosis, ferrostatin-1 was found to ameliorate lung injury through improving pulmonary oedema, inhibiting lipid peroxidation, and increasing epithelial cell viability. However, Fe was found to reverse the above changes. Thus, we conclude that ferroptosis occurs in IIR-ALI and OGD/R and exacerbates both lung injury and cell damage. Moreover, inhibition of ferroptosis protects against this lethal process.

As an essential transcription factor of oxidative responses, Nrf2 has been reported to activate the expression of various target genes that are indispensable for modulating ferroptosis by regulating the metabolism of glutathione, iron and lipids, and mitochondrial function [20]. In addition, activation of the Nrf2 pathway have been shown to promote the proliferation of cancer cells through blunting their responses to ferroptosis inducers [21, 22]. However, the intrinsic effect of Nrf2 on ferroptosis still remains to be investigated in IIR-ALI. In this study, we found that increasing expression of Nrf2 exerted resistance to ferroptotic injury in IIR-ALI models. In *Nrf2* knockout mice under IIR-ALI conditions, the expression of anti-ferroptotic factors (e.g., GSH and GPX4) was further decreased in contrast with that in the WT IIR group. These results indicate that an Nrf2 deficiency enhances IIR induced lung damage by promoting ferroptosis.

SLC7A11 is a specific light chain subunit of the cystine/glutamate antiporter, which acts as a negative regulator of ferroptosis by maintaining a steady state of redox [23]. Sun et al. [21] found that iron regulates the transcription of SLC7A11 through the ROS-Nrf2 pathway. Additionally, Activation of Nrf2 was found to up-regulate multiple ROS-detoxifying enzymes, including hemeoxygenase1 (HO-1) [24]. In this study, the expression of SLC7A11 and HO-1 were increased in a compensatory manner in IIR or OGD/R, which could be enhanced with the use of Fe or alleviated following the administration of Ferroportin-1. This indicates the involvement of these factors in IIR-ALI-associated ferroptosis. Furthermore, knocking down *Nrf2* could significantly decrease the level of SLC7A11 and HO-1 protein expression and facilitate the accumulation of lipid peroxide. These findings illustrate that Nrf2 may exert its anti-ferroptotic role by regulating SLC7A11 and HO-1 expression.

To further explore the relationship between SLC7A11 and HO-1, lentiviruses were used to overexpress or inhibit *SLC7A11*. Interestingly, SLC7A11 appeared to negatively regulate Nrf2/HO-1 signalling. Nrf2 and HO-1 were activated in this model due to intracellular ROS accumulation. Up-regulation of *SLC7A11* increased the intracellular level of cystine and alleviated oxidative stress in epithelial cells, which resolved the positive stimulus of Nrf2/HO-1 expression to some degree. Additionally, *SLC7A11* interference appears to significantly protect against cell death in OGD/R models as shown by the CCK8 results. In addition to its well-established antioxidant role, SLC7A11 is also an important metabolic regulator exerting the nutrient flexibility of cells. Under glucose-deficient or glutamine-replete conditions, the down-regulation of SLC7A11 markedly improves cell viability by enhancing the utilization of intracellular glutamate to maintain respiratory chain activity [25]. This phenomenon often occurs in tumour cells with glucose starvation. In this study, cells subjected to oxygen and glucose deprivation may be confronted with a similar situation, which makes SLC7A11 act as a double-edged sword in regulating the redox balance and nutrient dependency.

Further studies may need to be conducted to elucidate intrinsic mechanisms of the IIR ALI. Firstly, necroinflammation has been found to be involved in distant organ injury [26, 27]. The activated innate immune system has been reported to initiate local inflammation and subsequently lead to necroinflammation in remote organs via spreading damage-associated molecular patterns and cytokines to the systemic circulation from primary ischemic organs [27, 28]. In the present study, II/R induced ferroptosis may activate the innate immune system and lead to cytokine release, which ultimately cause necroinflammation in the lung. Secondly, ferroptosis-suppressor-protein 1 (FSP-1) was recently reported to be a key component of a non-mitochondrial antioxidant system. Moreover, it was proven to inhibit ferroptosis via preventing lipid oxidation [29, 30]. Further investigations are required since both Nrf2 and FSP-1 are closely related to CoQ antioxidant systems [31].

In conclusion, our study demonstrated the presence of ferroptosis in IIR-ALI. Nrf2 exerts a protective role in IIR-ALI through regulating ferroptosis by promoting the expression of HO-1 and SLC7A11.

## MATERIALS AND METHODS

### Experimental model

A total of 60 eight-week-old C57BL/6J mice and 48 eight-week-old *Nrf2* knockout (Nrf2^−/−^) mice with the same genetic background (provided by the RIKEN Bio-Resource Centre through the National Bio-Resource Project, MEXT, Japan) were used to conduct in vivo experiments. The mice were housed under controlled temperatures (21°C ± 2°C) and humidity (60% ± 5%) on a 12-h light/dark cycle. Mice were fed standard mouse chow and water ad libitum. All experiments were conducted in line with the NIH guidelines and approved by the Ethical Committee of Shanghai Ninth People’s Hospital for Animal Research.

### IIR mouse model

Mice were randomly assigned to four groups: (n = 6/group): 1) sham; 2) IIR; 3) IIR + Fe; and 4) IIR + Fer-1. The mice were then anaesthetized with sodium pentobarbital (50 mg/kg, i.p.) and permitted to breathe normally during surgery. The abdominal wall of the anaesthetized mice was opened with a midline incision. The superior mesenteric artery was exposed in the sham group without occlusion. Non-invasive vascular clips were used to block the superior mesenteric artery for 45 min in the IIR group and then recovered reperfusion was performed for 180 min to generate an ALI model of IIR. Prior to blocking the superior mesenteric artery, 15 mg/kg Fe-citrate(III) (Fe) (CAS 2238-05-8, Sigma-Aldrich, USA) and 1.5 mg/kg ferrostatin-1 (Fer-1) (CAS 347174-05-4, Sigma-Aldrich, USA) were injected into the tail vein in the IIR + Fe group and IIR + Fer-1 groups, respectively. All mice were resuscitated with an intraperitoneal injection of normal saline (1.0 mL) after surgery. Animals were sacrificed following 3 h of reperfusion and the tissues were harvested. Tissues were snap frozen in liquid nitrogen and stored at -80°C until further analysis.

### Histological examination

Ischemia-reperfusion injury was determined by analysing 4-mm haematoxylin and eosin (HE)-stained paraffin-embedded sections of the mouse lung tissue. A histological examination was performed as described in the Supplementary Materials.

### Pulmonary oedema

The level of pulmonary oedema was tested using a wet to dry ratio (W/D). Pulmonary oedema was performed as described in the Supplementary Materials.

### Cell culture

MLE12 pulmonary epithelial cells and Beas-2b human bronchial epithelial cell lines were purchased from the cell bank of the Chinese Academy of Sciences (Shanghai, China). These cells were cultured in DMEM (HyClone, USA) or BepiCM (ScienCell, USA) medium supplemented with 10% foetal bovine serum (GIBCO, USA), penicillin (100 IU/mL), and streptomycin sulphate (100 μg/mL) at 37°C in a thermostatic incubator containing 5% CO_2_.

### Oxygen-glucose deprivation (OGD) and reoxygenation model

To create a model of oxygen-glucose deprivation and reoxygenation (OGD/R), the cells were cultured in glucose-free DMEM (TBI; China). The cells were washed in PBS supplemented with 0.5 mM CaCl_2_ and 1 mM MgCl_2_ and placed in an anaerobic chamber (5% CO_2_, 95% N_2_; Memmert; Schwabach, Germany) to induce OGD. After 8 h, the medium was replaced with normal culture medium and the plates were incubated in a normoxic chamber (37°C, 5% CO_2_) for 12 h of reoxygenation.

### Cellular proliferation assays

Cellular proliferation was estimated using a Cell Counting Kit-8 (Dojindo, Kumamoto, Japan) assay. Cellular proliferation assays were performed as described in the Supplementary Materials.

### RNA extraction and real-time PCR (RT-PCR)

The total RNA was isolated using TRIzol reagent following the manufacturer's protocol (Life Technologies) and subjected to cDNA synthesis using a Prime Script RT-PCR kit (TAKARA Korea, Seoul, Korea). A real-time RT-PCR analysis was performed using SYBR mix with a CFX384 real-time system (BioRad, Hercules, CA, USA). The amplification protocol was comprised of the following PCR cycles: a single cycle of 5 min at 95°C, 40 cycles of 10 s at 95°C, 10 s at 59°C, 20 s at 72°C, and a final cycle of 10 s at 95°C. The following real-time PCR primers were used in the present study: Nrf2 upstream: 5’-TAGAGTCAGCAACGTGGAAG-3’ and downstream: 5’-TATCGAGGCTGTGTCGACTG-3’; SLC7A11 upstream: 5’-GCTGACACTCGTGCTATT-3’and downstream: 5’-ATTCTGGAGGTCTTTGGT-3’; HO-1 upstream: 5’-TAGAGTCAGCAACGTGGAAG-3’ and downstream: 5’-TAGAGTCAGCAACGTGGAAG-3’.

### Preparation of nuclear extracts

Nuclear extract preparation was performed as described in the Supplementary Materials (Supplemental Digital Content 1).

### Western Blot (WB) analysis

Western blotting was carried out using a standard protocol. Proteins (30 μg) in the total cell lysates from the lung tissue were separated by SDS-PAGE and transferred to PVDF (polyvinylidene fluoride) membranes. WB was performed using the following primary antibodies: Nrf2 (ab137550, Abcam, 1:1000); SLC7A11 (ab37185, Abcam, 1:1000); HO-1 (70081, Cell Signaling Tech, 1:1000); GPX4 (ab125066, Abcam, 1:1000); and β-Actin (4970S, Cell Signaling Tech, 1:1000). All Bands were visualized by chemiluminescence (Millipore, USA), followed by exposure to x-ray film (RX-U; Fujifilm) and densitometrically quantified using ImageJ software (National Institutes of Health). (Supplementary Materials).

### RNA interference and gene transfection

To overexpress or inhibit related genes, M_SLC7A11-shRNA (PGMLV-SC5); M_Nrf2 (NFE2L2)-shRNA2 (SB3); CMV-M_SLC7A11-3 × Flag-PGK-Puro and CMV-M_Nrf2-3 × Flag-PGK-Puro lentiviral vectors were purchased from Genomeditech (Shanghai, China). The lentiviral vectors were transfected into MLE12 and Beas-2b cells, and the Puro Lentivirus and Scramble-SB3 Lentivirus were used as negative controls. For the stable silencing and over-expression of the Nrf2 or SLC7A11 genes, the cells were plated into six-well plates at a density of 2.5 × 10^5^ cells/well and allowed to adhere overnight. The following day, the cells were transfected with lentiviruses using the manufacturer’s protocol. Stably transfected cells were selected using 2 μg/mL puromycin (1299MG025, BioFroxx, Germany). The efficiency of silencing was confirmed by a WB and qRT-PCR after 72 h of transfection.

### Measurements of malondialdehyde (MDA) and glutathione (GSH) levels

The level of MDA and GSH were determined using a Lipid Peroxidation (MDA) Assay Kit (MAK085; Sigma-Aldrich) and an activity kit (Nanjing Jiancheng Bioengineering Institute, China), respectively.

### Transmission electron microscopy

The lung tissues were fixed for 2 h with 2.5% glutaraldehyde in a 0.05 M sodium cacodylate buffer at a pH of 7.2 at 25°C, followed by 2 h in 2% OsO4 in a 0.1 M sodium cacodylate buffer and 18 h in 1% aqueous uranyl acetate. After dehydration through an ethanol series, the specimens embedded in Epon 812 and ultrathin sections were collected on copper grids. After staining with uranyl acetate and lead citrate, the sections were examined using a Tecnai G2 spirit BioTwin transmission electron microscope (FEI Company, Hillsboro, Oregon).

### Statistical analysis

SPSS 22.0 statistical software (SPSS, USA) was used to analyze the experimental data. All data were presented as the mean ± SD (standard error of the mean). Data were compared using a one-way ANOVA. *P* < 0.05 was considered to indicate statistical significance.

## Supplementary Material

Supplementary Materials

Supplementary Figures
