# Correction for: Nrf2 inhibits ferroptosis and protects against acute lung injury due to intestinal ischemia reperfusion via regulating SLC7A11 and HO-1

**DOI:** 10.18632/aging.205167

**Published:** 2023-10-14

**Authors:** Hui Dong, Zhuanzhuan Qiang, Dongdong Chai, Jiali Peng, Yangyang Xia, Rong Hu, Hong Jiang

**Affiliations:** 1 Shanghai Ninth People’s Hospital, Shanghai JiaoTong University School of Medicine, Centre for Specialty Strategy Research of Shanghai Jiao Tong University China Hospital Development Institute, Shanghai 200011, China

**Keywords:** ferroptosis, acute lung injury, solute carrier family 7 member 11, heme oxygenase-1, nuclear factor erythroid 2 related factor2

**This article has been corrected:** The authors found an error in the **RNA extraction and real-time PCR (RT-PCR)** section of the **Materials and Methods**, where primers were replaced with the correct *Mus musculus* sequences:

“… The following real-time PCR primers were used in the present study: Nrf2 upstream: 5’-GATTCACGCATAGGAGCACTG-3’ and downstream: 5’-CTTCCATTTACGGAGACCCAC-3’; SLC7A11 upstream: 5’-GCTGACACTCGTGCTATT-3’and downstream: 5’-ATTCTGGAGGTCTTTGGT-3’; HO-1 upstream: 5’-AGACCGCCTTCCTGCTCAACAT-3’ and downstream: 5’-TCTGACGAAGTGACGCCATCTGT-3’

Additionally, the authors want to clarify the overlap in **Figures 1C** and **2E**, which show HE staining of lung tissues from mice following IIR, IIR + Fe, and IIR + Fer-1 treatment. The same image of sham HE staining was used in both figures, which is now acknowledged in the legend to **Figure 2E**:

**Figure 2. Nrf2 regulates SLC7A11 and HO-1 to inhibit ferroptosis and protect against IIR-ALI. **… (**E**) Representative HE-stained lung sections. Morphology was examined using light microscopy. Scale bars: 200 μm. The image of Sham group is the same as the one of Sham group in Figure 1C. …

The Authors also found that the western blot bands for Nrf2 and HO-1 from **Supplementary Figure 2A** were accidentally misused in **Supplementary Figure 3A**. They prepared a new **Supplementary Figure 3A** using images from the original experiments and recalculated the relative densities of these proteins.

These corrections do not impact the overall findings and conclusions of the paper. The authors would like to assure readers that the corrected values do not alter the interpretations or validity of the research and they apologize for the errors.

New **Supplementary Figure 3A** is presented below.

**Supplementary Figure 3:**
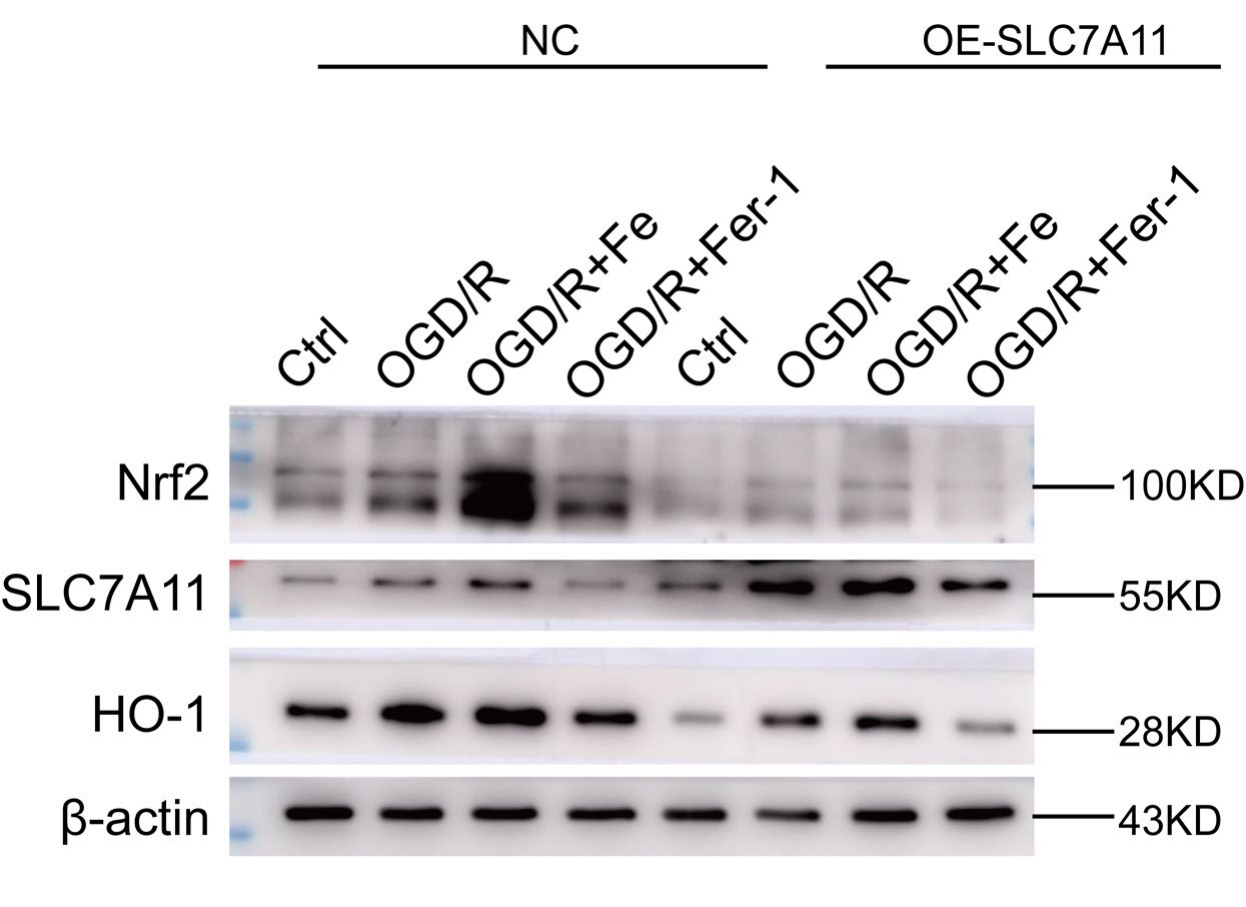
**Low levels of SLC7A11 alleviate cell death by upregulating Nrf2-HO-1, whereas SLC7A11 overexpression (OE-SLC7A11) enhanced cell death.** (**A**) SLC7A11 overexpression downregulates Nrf2-HO-1. Western blot showing the level of protein expression in BEAS-2B cells.

