# Supplementary material for: Nrf2 inhibits ferroptosis and protects against acute lung injury due to intestinal ischemia reperfusion via regulating SLC7A11 and HO-1
Source: Aging (Albany NY). 2020 Jun 29;12(13):12943–59. doi: 10.18632/aging.103378 (PMC7377827; doi:10.18632/aging.103378)
Supplement: Supplementary Materials [file aging-12-103378-s001..pdf]

SUPPLEMENTARY MATERIALS

MATERIALS AND METHODS

Animals

All animal procedures were approved by the Ethical Committee of Shanghai Ninth People's Hospital for Animal Research.

Cell culture

Mice lung epithelial MLE12 cells and Human lung epithelial BEAS-2b cells were purchased from the cell bank of the Chinese Academy of Sciences (Shanghai, China). These cells were cultured in DMEM (HyClone, USA) or BepiCM (ScienCell, USA) medium supplemented with 10% foetal bovine serum (GIBCO, USA), penicillin (100 IU/mL), and streptomycin sulphate (100 μg/mL) at 37°C in a thermostatic incubator containing 5% CO_2.

Histological examination

Ischemia-reperfusion injury was determined by analysing 4-mm haematoxylin and eosin (HE)-stained paraffin-embedded sections. ALI was scored using a five-point scale according to combined assessments of alveolar congestion, haemorrhage, oedema, and inflammatory cell infiltration in the airspace or vessel wall. A 0–4 (minimal to maximal severity) scoring system was used in a blinded manner (1). Images were captured with a digital camera (Optronics DEI-470; Goleta, CA) connected to a light microscope (Nikon-Ni-U; Japan).

The degree of lung injury was assessed using a scoring system in which the level of oedema of the alveoli and mesenchyme, intra-alveolar inflammatory cell infiltrates, alveolar haemorrhage and atelectasis were graded on a scale between 0 and 4. The grades were as follows: 0, normal, < 15% of space is occupied by tissue and > 85% occupied by alveolar space; 1, 15%–25% of space is occupied by tissue and 75%–85% is occupied by alveolar space; 2, 25%–50% is occupied by tissue and 50%–75% is occupied by alveolar space; 3, 50%–75% is occupied by tissue and 25%–50% is occupied by alveolar space; and 4, 75%–100% is occupied by tissue and 0%–25% is occupied by alveolar space.

Pulmonary oedema

The level of pulmonary oedema was tested using a wet to dry ratio (W/D). A median sternotomy was performed after completing reperfusion. The lung lobe was cut from the pleural cavity and the right lung was placed in a drying oven (90°C) for 72 h after it was weighed. The right lung was weighed again after drying was complete. The wet weight of the lung was divided by the dry weight to calculate the wet to dry ratio. The middle lobes of the right lungs were weighed (weight wet) immediately using a precision balance (Mettler-Toledo, Schweiz, GmbH, Greifensee, Switzerland), and re-weighed (weight dry) following an incubation at 95°C in an oven (876–1 Vacuum Drying Oven; Nantong Science Instrument Factory, Nantong, China) for 72 h. The W/D was calculated using the following equation:

W/D = weight wet/weight dry.

Proliferation screening and cellular proliferation assays

The cellular proliferation activity was estimated using a Cell Counting Kit-8 (Dojindo, Kumamoto, Japan) assay. To measure the proliferative activity of cells in 96-well microplates, CCK-8 was added (10 μL/well) and incubated for 2 h. Absorbance was measured at 450 nm using a microplate reader (Molecular Devices) with a reference wavelength of 650 nm.

Preparation of nuclear extracts

Nuclear extracts were prepared in accordance with a previously described method [19]. Briefly, the cells were collected by scraping in ice-cold PBS containing phosphatase inhibitors and pelleted by centrifuging at 1000 × g for 5 min. The pellet was resuspended in 500 mL of 1 × hypotonic buffer and incubated on ice for 15 min, followed by the addition of detergent (25 mL) and high-speed vortexing for 30 s. The suspension was then centrifuged (14,000 × g for 20 min) at 4°C. The nuclear pellet was resuspended in lysis buffer (50 mL) and incubated on ice for 15 min. This suspension was centrifuged (14,000 × g) for another 10 min and aliquots of the supernatant (nuclear extract) were stored at –80°C until further analysis. The protein content in the nuclear extract was quantified by a BCA protein assay.

Western blotting (WB) analysis

Western blotting was carried out using a standard protocol. Proteins (30μg) in the total cell lysates or lung tissue were separated by SDS-PAGE and transferred to PVDF (polyvinylidene fluoride) membranes. The membrane was immersed in blocking buffer for 1 h before incubation with primary antibodies overnight at 4°C. The primary antibodies are: Rabbit monoclonal anti-Nrf2 (ab137550, abcam, 1:1000); anti-SLC7A11

(ab37185, abcam, 1:1000); anti-HO-1 (70081, cell signaling tech, 1:1000); anti-GPX4(ab125066, abcam, 1:1000); anti-β-Actin (4970S, cell signaling tech, 1:1000). After rinsing in wash buffer, horseradish peroxidase-conjugated secondary antibody was used for 1 h at room temperature. After the final wash, the membrane was developed using ECL Reagent, and densitometric analyses were conducted using Image J Software. Bands were visualised by chemiluminescence (Millipore, USA), followed by exposure to x-ray film (RX-U; Fujifilm) and densitometrically quantified using Image J software (National Institutes of Health).

REFERENCE

1. Yan J, Li J, Zhang L, et al: Nrf2 protects against acute lung injury and inflammation by modulating TLR4 and Akt signaling. Free Radic Biol Med 2018; 121:78–85 https://doi.org/10.1016/j.freeradbiomed.2018.04.557 PMID:29678610
