# Supplementary material for: Nrf2 inhibits ferroptosis and protects against acute lung injury due to intestinal ischemia reperfusion via regulating SLC7A11 and HO-1
Source: Aging (Albany NY). 2020 Jun 29;12(13):12943–59. doi: 10.18632/aging.103378 (PMC7377827; doi:10.18632/aging.103378)
Supplement: Supplementary Figures [file aging-12-103378-s002..pdf]

SUPPLEMENTARY FIGURES

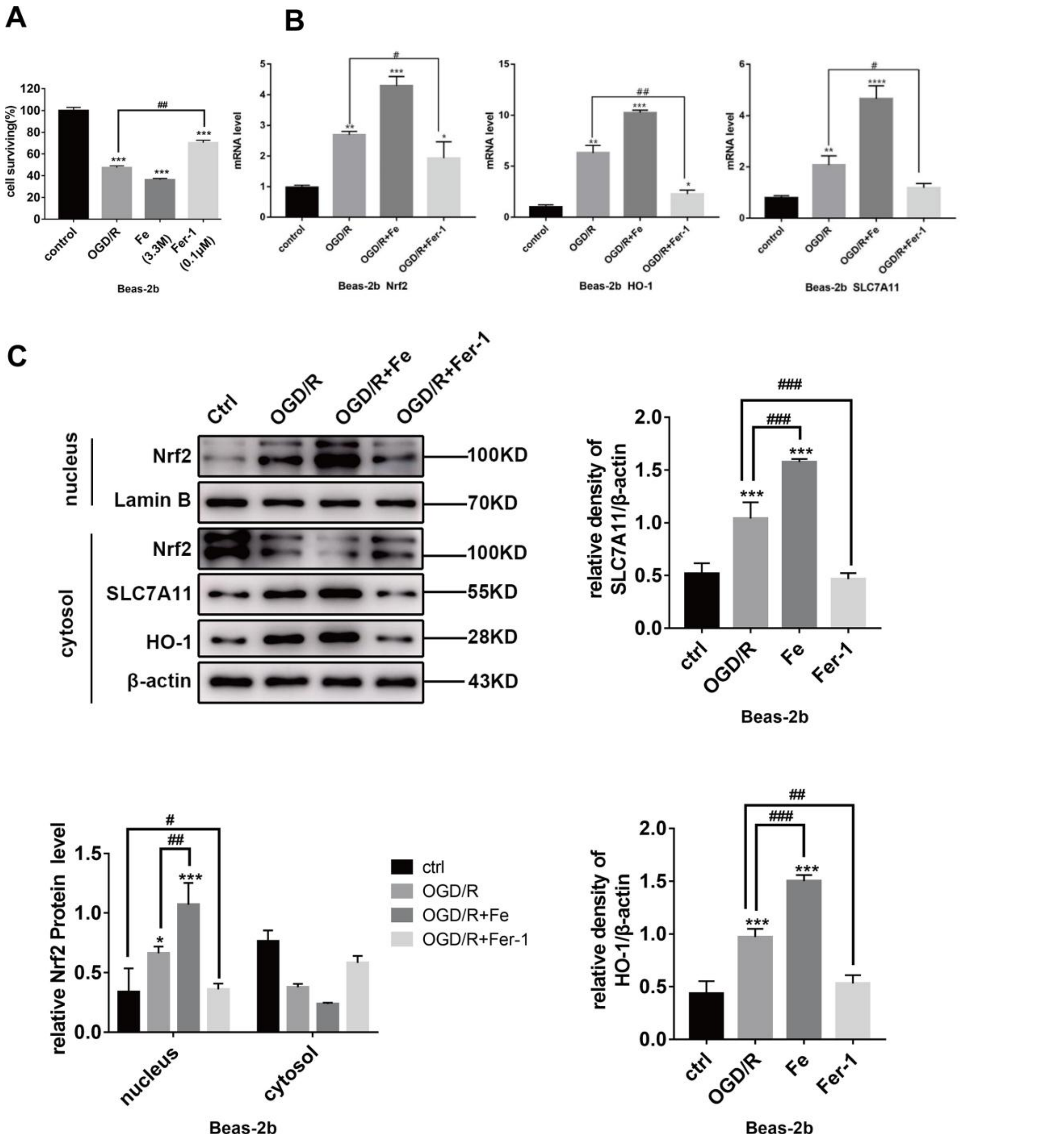

Supplementary Figure 1. OGD/R induces ferroptosis in human pulmonary epithelial cells and increases the level of Nrf2 /SLC7A11/HO-1 expression during ferroptosis. (**A**) The cells surviving after OGD (8 h)/R (12 h), while the administration of Fe (3.3M) (800 μg/mL)/Fer-1 (0.1 μM) can respectively increase or decrease the ratio. (**B**) Fer-1 prevented the increase in the level of HO-1 and SLC7A11 mRNA expression after OGD/R, and Fe promoted the increase of Nrf2, HO-1, and SLC7A11 mRNA expression in Beats-2b. (**C**) Western blotting of Nrf2, HO-1, and SLC7A11, and the representative quantification of these three proteins. The level of protein expression was increased in OGD/R, which was exacerbated by Fe and prevented by Fer-1. The error bars represent the standard error from three replicates. *$P < 0.05$; **$P < 0.01$; ***$P < 0.001$ between the groups; *compared with control; # compared with OGD/R.

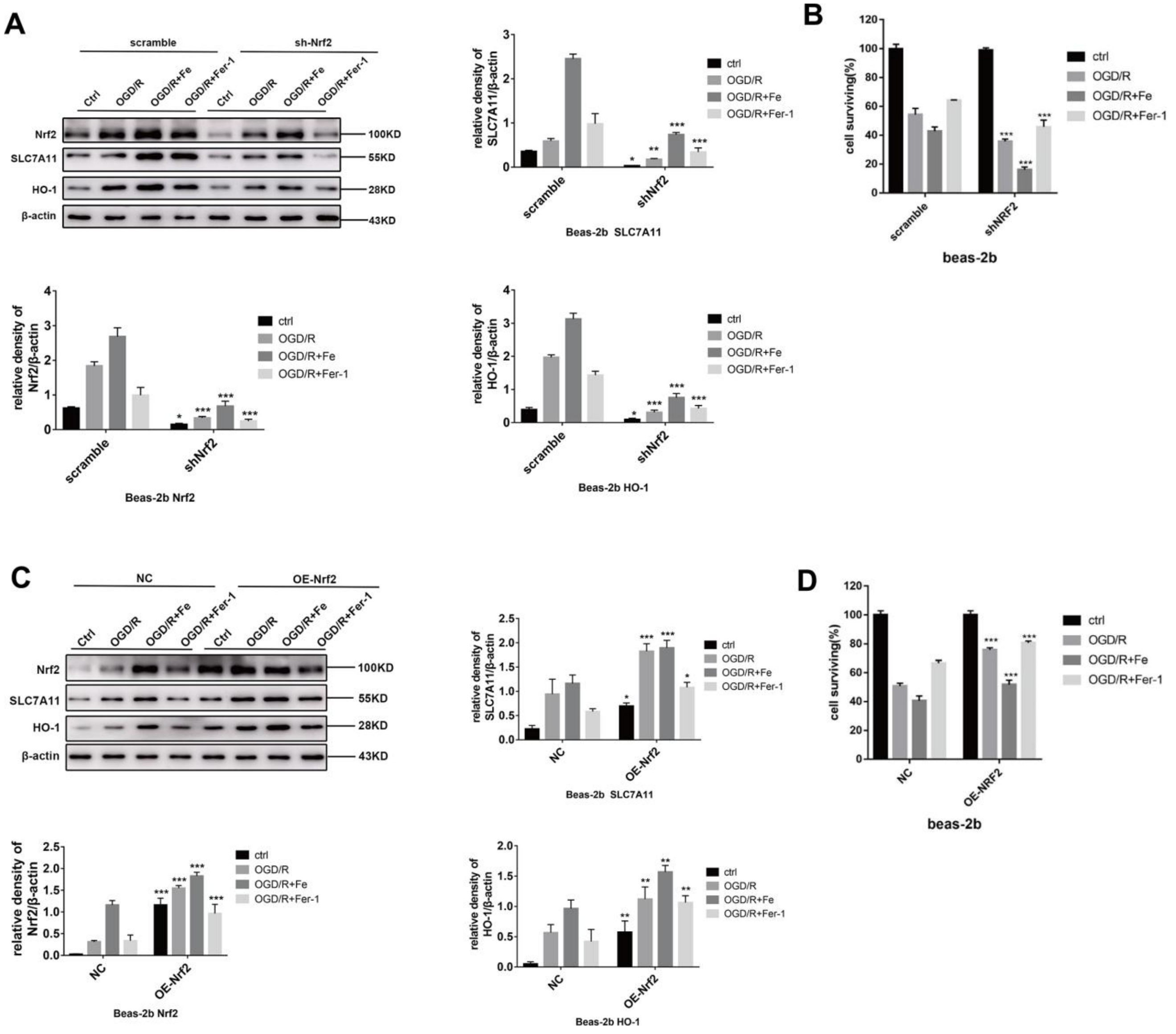

Supplementary Figure 2. Nrf2 activation contributes to ferroptosis resistance. The Beas-2b cells were transfected with lentiviruses. (**A**) Compared with the scrambled vector, the level of SLC7A11 and HO-1 protein expression was significantly reduced after transfection with an NRF2-shRNA lentivirus, but the expression changes of these factors after Fe or Fer-1 administration were consistent with those in the scramble group in Beas-2b. (**B**) The cells that survived after OGD (8 h)/R (12 h). Compared with the scrambled group, cell survival was lower after the knockdown of Nrf2. The OGD/R + Fe group was also more severe than the corresponding NC group. Lung injury was alleviated after treatment with Fer-1. (**C**) The level of SLC7A11 and HO-1 protein expression was significantly increased after transfection with an Nrf2 overexpression lentivirus compared with the group transfected with the control vector, but the changes in the expression of these factors after Fe or Fer-1 administration were consistent with those in the NC group in Beas-2b. (**D**) The cells surviving after OGD (8 h)/R (12 h). Compared with the scrambled group, there was enhanced cell survival following the overexpression of Nrf2. The error bars represent the standard error from three replicates. The error bars represent the standard error from three replicates. *P < 0.05; **P < 0.01; ***P < 0.001 between the groups. *compared with control.

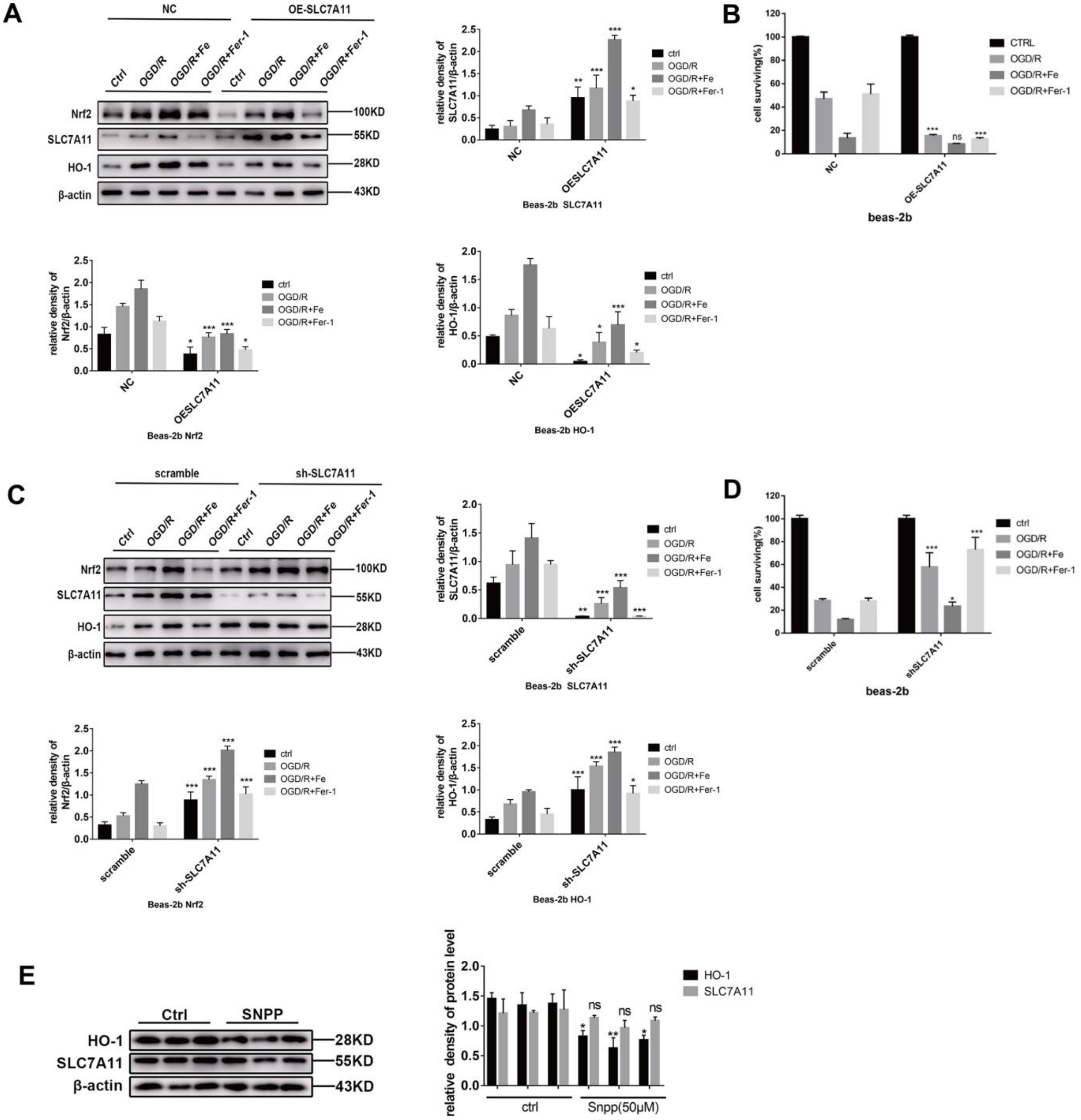

Supplementary Figure 3. Low levels of SLC7A11 alleviate cell death by upregulating Nrf2-HO-1, whereas SLC7A11 overexpression (OE-SLC7A11) enhanced cell death. (**A**) SLC7A11 overexpression downregulates Nrf2-HO-1. Western blot showing the level of protein expression in BEAS-2B cells. (**B**) SLC7A11 overexpression promoted cell death. (**C**) SLC7A11 interference upregulates Nrf2-HO-1 protein expression in Beats-2b. (**D**) SLC7A11 interference alleviated cell death. (**E**) SNPP (HO-1-specific inhibitor) administration inhibited HO-1 expression, whereas SLC7A11 expression had no significant effect. The error bars represent the standard error from three replicates. **P* < 0.05; ***P* < 0.01; ****P* < 0.001 between the groups. *compared with control.
